# Professional Patents

**Published:** 1888-12

**Authors:** B. Holly Smith

**Affiliations:** Baltimore


### Independent Practitioner.

VOL. IX. DECEMBER, 1888. NO. 12.

NOTE.—No paper published or to be published in another journal will be accepted for this department. All papers must be in the hands of the Editor before the first day of the month preceding that in which they are expected to appear. Extra copies will be furnished to each contributor of an accepted original article, and reprints, in pamphlet form, may be had at the cost of the paper, press-work and binding, if ordered when the manuscript is forwarded. The Editor and Publishers are not responsible for the opinions expressed by contributors. The journal is issued promptly, on the first day of each month.

### Original Communications.

### PROFESSIONAL PATENTS.

BY B. HOLLY SMITH, BALTIMORE.

READ AT THE EIGHTEENTH ANNUAL MEETING OF THE NEW JERSEY STATE DENTAL SOCIETY, HELD AT ASBURY PARK, JULY 18, 19, 20, 1888.

Of all the questions which we have need to discuss impartially and decide wisely, there are probably none outranking in importance the question, "Should a dentist patent his inventions?" It is not a question, probably, the discussion of which, either one way or the other, will seriously impede or affect the material prosperity and progress of the profession. In this dawn of the 20th century, the spirit of scientific investigation is so rife and so wide awake, that a question of patents or no patents in any given calling is not apt to stay its inquiries or lessen the number or magnitude of its discoveries.

Neither dearth nor plenty of patents can stifle science; for science and scientific thought were before patents, and will be after they have faded from the earth, if ever that unpractical and un-American period comes.

The foundation of dentistry is laid broad and deep in the supporting substructure of scientific principles. But strange as it may appear in this age of a regal and imperial materialism, there is another standpoint from which we must view our profession and from which we must judge what is to its best interests, what best comports with its true elevation, and that we may say is the ethical standpoint. It is well for us that we can come to the discussion of this question in the peculiarly favorable circumstances of our times and surroundings—with the examples of older professions to guide us, and the free thought of younger minds ready to give potent arguments for new courses—with the experience and judgment of an older order of things, and the ardor and courage of youth and its attendant spirit of audacity, innovation and investigation. A judicious mingling of these elements of conservatism and desire for change should set us aright in our discussion and decision of this great and interesting question. First, then, let us review briefly the example and experience of medicine in this matter; examining at the same time, if we can, the reasons at the base of its course, and the effect of such a course on the progress of medicine.

The position of medicine is flatly opposed to patents among the members of its profession, and medical ethics brands it as an unprofessional action for anyone bearing an M.D. after his name to take out a patent for a medical or surgical invention. To those of us who so strictly insist on recognition as a branch of that noble and time-honored profession, it seems this should be an all sufficient reason for a similar decision on our part. Those who become members of a family or society by birth or by adoption cannot expect to alter its established traditions and laws, while composing but a small part of its body politic in numbers or influence. They must therefore accept the regimé under which they have been received, until their weight and influence are sufficient to change it; and to all dentists who rejoice in their kinship to the noble science of medicine I would say whether you have taken up medicine or medicine has taken up you; in either case, the ethics of your profession must be the ethics of medicine. Your claim, if there be any among you who have such a claim, to the warrantableness of patents in your profession, is summarily dismissed by a reference to the code by whose principles you must be governed or to whose practice you must prove recreant.

But many of us are not content with so large a use of authority and so small an argument. The desire exists that reasons be submitted to fair and candid judgments, untrammelled, if possible, by prejudice, uninfluenced by that pride of position which accepts professional traditions with unthinking submission. To such we think a strong appeal can be made, which the franker and better natures will not cast away unheeded. The first reasons for which I think it unprofessional for dentists to patent their inventions is based on a view of the position which our profession occupies towards the rest of mankind, as a dispenser of blessings and comforts to all who come under its enlightened and beneficent healing powers. A high conception of the mission of our profession is not only important in the discussion of all questions pertaining to it, but must be the essential element of that discussion, its soul and vitality, else the poverty of degeneration will encumber us with the festering putridness of professional degradation, charlatanism and quackery. With the most settled conviction we hold the belief that our mission is among the noblest of all professions ; and if this belief be deception, I do not desire to be undeceived; but will discuss the question from this vantage-ground. Shall we be told that the profession to which we have given the ardor of youth, the maturity of manhood, and to which we hope to add the experience of age, with fervor, constancy, and consistency—that we regard it in no higher light than the earner of our bread ; no higher than the donkey to toil before the cart of self-profit, whose hide and bones we would be glad to sell when he can no longer serve us. Shall it be said that we practice dentistry solely for what we can get out of it?

This is what is called a practical age, and I may be talking to practical men, men who gauge their success by the number of figures in their bank balance ; but I refuse to credit, wherever announced the statement that the men of our profession would so mistreat and so misconcieve the mission of dentistry as to deal with her solely as the channel through which the dollars are to run from the great public to the individual practitioner, to which main-pipe each professional brother would open his little side pipe and by dint of scooping and scratching from the main and from his brothers' ducats strive to increase his pile. If, then, our mission as professional men does not have money getting for its sole aim; if it is our desire and purpose to alleviate the sufferings and promote the health and happiness of the human race, we should certainly take the humanitarian view and enrich the profession by the result of our labors, extend its sphere of usefulness and honor, learn that the advancement of the profession is more than the profit of the individual, and be content with that honor which dentistry hastens to pay her brainy and unselfish sons. If my brother has been fortunate enough to make a discovery by which the pain of thousands may be lessened, why should he not rejoice to make known his discovery to his thousands of brethren that the dominion of misery and disease may be abridged? Why does he withhold his benefit with suspicious eye and reluctant hand in order to reap a handsome profit from pain ridden humanity? And yet my brother would fain acquit himself of the monstrous charge of trading in human suffering. He would evade the issue by calling on his brother dentists to pay him for the product of his brains, forgetting that the increased cash of his bonus on his patented invention does not fall on that professional brother, but on the ultimate beneficiary.

All that is known to the profession my brother has the advantage of. Does he refuse to contribute his share to that knowledge and practice which enable that profession to accomplish its mission more readily and effectively? Does he cavil amid the sighs and groans of an afflicted humanity about the price he has paid for his professional knowledge? Can he ever form a cash estimate of the value of those services which the unrewarded and unthanked philosophers, the pioneers, the explorers and subjugators of science have given to truth, to investigation and to him?

We have been told that our superiority of vocation is assumed. That no services can be performed for mankind for which it cannot and does not desire to pay. That all men in this peculiar respect are equal before God, all are kings; but we confess that we see no kingly attribute in this indecent clamor, not for honor, not for glory or renown, but for pelf. Kingliness and munificence, royalty and honor, regal plenty and abundant gifts. We have associated; but to haggle and chaffer for prices and payments: these are not the acts of nature's monarchs.

There are few of us who have not learned at least some of the blessings of charity and of love; few who have not willingly performed services for which the only compensation could be the proud consciousness of the good done. Enlarge such a sphere of usefulness and let the thought that it is not one or a few to whom the beneficence of your labor goes, but to the human race, wherever dentistry is practiced; and if that thought does not possess a supreme comfort and a grandeur for you, your faith in the brotherhood of man is truly weak. Still, to some such a reward may be too intangible. The faculty, which can gather and enjoy the ever accumulating interest of disinterestedness, may be lacking in them. To those I would say it is your duty to use and not to abuse the position which you have not acquired yourself, but which, under God, your profession has given you; and all you do for your fellow man in increasing that profession's general effectiveness and power is consecrated work.

The legitimate, honorable and professional practice of dentistry has brought affluence to some and a livelihood to all who have united ability and application. Yet I do not think there is a man among us who would not forego the just reward of his labor rather than do aught to retard the development of our calling. I will go a step further, and say that the thought that he is adding something to the profession and assisting to project it along its destined course of usefulness and honor would recompense any true dentist for a slighter return for his professional toil and exertions.

Yet does the gift of an invention to the profession make the inventor poor? Only in the negative sense, that it has made mankind more comfortable, added another agency to his profession's powers for work and relief, and augmented his professional brother's equipment. The inventor is no whit poorer; but the world and the profession are richer.

After all, just as humanity is greater than the profession, so is the profession greater than its individual practitioner. It represents the aggregate of the knowledge and skill which forms the equipment of its members. It embodies the science and research whenever and wherever made, which render that knowledge and skill possible. It has seized upon and assimilated, with no royalty, to those patient investigators to whom the truths of nature have been revealed, the results and conclusions of those revelations by mere virtue of its kingly office as healer and curer. The normal function of each member of the profession is to help augment its knowledge and increase its skill. In soberness and truth he should remember that he gets much and gives little. What we want to-day is the scientific spirit—the spirit which, like Chaucer's noble student, will gladly learn and gladly teach. The spirit which inquires, investigates, invents, discovers and scatters the results broadcast to the world; the spirit which has done more for dentistry as a daughter and descendant of science than all the selfish stimulus of patents ever will or ever can do. Can those who patent their inventions in a profession be said to possess this spirit? They lack its essence and vitality. Not only do they not add to the sum total of professional knowledge, but they subtract from it by stunting and dwarfing its more general growth. Just as patent medicines have made it possible for quackery and charlatanism to thrive, so will patent dentistry foster stagnation among dental professional activities. The weaker brethren, instead of either becoming stronger or going to the wall, as they ought, will hang on to reflect discredit upon dentistry by a lame manipulation of labor-saving patents. For the progress of any profession depends more on the thorough study of that science which lies at its base than on a helpless and impotent reliance on toil-saving contrivances, alike enervating to the invention and energy of the poor dependents. If it be said that the free gift of inventions will produce a yet greater degree of enervation and lassitude, I respond no. Patents stifle general exercise of professional inventive genius, not because of the invention, but because of the patent. It becomes the policy of a successful and powerful business to push its patents to the supplanting of other inventions—a few whales swallow all the rest of the finny tribe, and the very device looked upon to foster and encourage inventive talent has the opposite effect.

Have surgical devices been fewer, and have other branches than dental surgery suffered by the exclusion of the patent system from their practice? Free discussion, candid criticism, and unpaid, well-merited praise have served the student in surgery well. The medical and scientific world are looking with impartial eyes upon his efforts. What he does that is good is approved and adopted; while with the approval there is a flavor of brought-up opinions, no smack of a well-disguised advertising scheme; but the frank judgment of a brother student, who has learned something in his chosen work, and is thankful for inspiration and strength. If in the business and trade world the supreme and only stimulus be selfishness and a hankering after the almighty dollar, it were surely next to crime to introduce so niggardly, cut-throat a policy into a profession.

Are we not one society whose courteous aim among ourselves is to help each other, or does each regard the other as his legitimate prey, upon whom to work off his little scheme, or to whom to sell his little patent? I know I need not hesitate for an answer to such questions in this body of men. The *esprit de corps* is as strong in dentistry as *in the other* learned professions, and will stand a yet greater strain than the patent system so long as the *personnel* of her membership feel so instinctively the honorable promptings of gentlemen and of brethren.

We have seen, then, that the experience and practice of the older profession proper is unqualifiedly against patents by its members, and that some of the reasons for this opposition are: From the position—a branch of the great healing art—occupies towards the world, having in its elevated and noble mission a real, and not an assumed, superiority of vocation; from the lack of harmony which the patent system in a profession exhibits with the true spirit of scientific investigation and inquiry; its tendency to stunt and dwarf the standard of eminent professional attainments, and to substitute for those requirements an easy-going charlatanism and a miserable quackery; the subjection of professional methods and instincts to business habits and customs; the submission to trade theories and trade valuations of services and acquirements which are not the subject of trade. We have seen that the stimulus which it offers is at *best* purely sordid, selfish and mercenary; that it cannot be reconciled with a refined and elevated *esprit de corps*, but consorts with the cut-throat, devil-take-the-hindmost policy of the mercantile world, with its advertising sufficient to plaster the globe over, its tons of samples, and its hundreds of thousands of iron-jawed drummers.

What are the arguments of those who support the patent system? They are excellently stated by Dr. W. Stoner How, of the S. S. White Dental Manufacturing Company, of Philadelphia, in a paper read before the Mississippi Valley Dental Association, in March of the present year. This paper is certainly well-written, nor can we think that better arguments, better put, can be produced for that side. It would seem, however, from our point of view, that the discussion is cast in a low key. The pro-patent advocates are no sooner in the field than they make a bee-line for their legal bulwarks, and set up a vigorous outcry about theprotection afforded their property in all the courts, both of law and of equity. Thus, at the outset, they shift the ground of contest, and evade the point at issue, which is as to its being professional or not for a dentist to patent his inventions.

The position of the pro-patent upholders, in a legal point of view, is impregnable. We shall not attempt to assail it. We certainly know that a patent is property, and that the law protects a man in the enjoyment of his property. Our friends have ably defended a point about which there has never been the slightest dispute, and though they have wasted much ammunition under the impression that they were about to be assailed, they have done themselves the credit of showing considerable legal lore and forensic acumen.

Why should a dentist go into a convention of dentists in a discussion like this and talk about the law of the patent system? Have we either to make or construe the law on that or on other points when the law touches us? Is not this matter troublesome enough as it is, without any legal complexities?

We grant all they demand as to property right and court protection. What then? The question we argue and the principle we contend for is to be settled right here among ourselves. Patent-rights, "the most unassailable rights to property on earth," will not take to themselves wings and fly away if it should happen to be a settled conviction in the minds of the weight of the dental profession that patents are unprofessional. So let us discuss the question here from a professional standpoint, in an amiable, dignified manner, and leave to the legal luminaries, truth-twisters and pettifoggers the drawing of nice distinctions between truth and falsehood, theft and profit, etc.

If the pro-patentees had continued their researches through the antiquated and fanciful Blakstone to the modern and philosophic Austen, they would have discovered that the law does not regard the ethical quality of the action it passes on. Its sole inquiry is; Have any published or established dictates been violated? When that proposition has been legally proved punishment follows: when not legally proved, acquittal is the result. Now, as the sole points at issue are the moral and ethical qualities of the discussed course of conduct, we cannot see that the law which would protect our brothers in court can have even the slightest weight here, since it omits the issue itself.

If there is one thing that exceeds the pro-patentees' deep sense of security behind his legal fastnesses, it is his admiration for business methods, business morals and business ethics. These combined form a code of Spartan simplicity, whose first commandment is, "Get all you can;" and the second, "Keep all you get." Most of us have experienced the elevation of business methods; but hear this outbust of enthusiastic wonder by the pro-patent advocate before mentioned, Dr. How:

"There is among men no higher working standard of honor than that of the business man, whose simple statement or working formula is: 'I will sell this to-day for so much;' and when his fellow-merchant, at hand or a thousand miles away, replies, 'I will take it at your price,' that practically completes the transaction."

As to this standard of honor, we have only to say that, in addition to the truly Christian spirit of forbearance which these merchants manifest in not lying to each other, there remains the cold, hard fact that a contract exists between them, which, honor or no honor, the somewhat firm and compulsive grip of the law will enforce, or punish the breach thereof. We see how slippery a customer our business man must be, that his simplest agreements in a business way are thus hedged about and enforced by this cold, unemotional body of law.

The standard is a pretty one; not below that of any honest man, and recommended at once by honesty and policy. We are led to suppose that there must be some cause for this legal support of the standard, and that some business men at least do not measure up to it with moral exactness. However well the standard may do to buy and to sell by, it can be of no use to us at present; and it pitches the key of discussion yet lower than the legal standard by which it has to be bolstered up. We cannot and will not assume that there is naught in our profession save what may be bought and sold—and if we ever do adopt the motto of Charles I, that "Every man has his price," I trust with splendid folly we shall prefer even empty honors, such as the gratitude of humanity, the esteem and reverence of our professional brethren, as the price paid us for our inventions and brain-products, rather than with business-like instinct to sell for hard cash not only our professional services, but our sense of honor.

Not only are the tests by which our patenting brethren would decide the professional quality of their action unfit criteria, but the motives which they impute to all who oppose the patent system are singularly sordid and impure. They not only simply but openly assert that all the talk about professional honor is naught but the high flown courtesy of a highwayman. A subterfuge which would enable the poor patentee to be robbed of the result of his labors, that the pockets of his brother dentists might be lined. In other words our pro-patent friends deliberately close their eyes in this debate to every view of the subject except the narrowest and most selfish. Having openly avowed their principal defense to be the legal right which they have to their property, having utterly ignored the claim of humanity and the profession, having set up the principle of barter as the basis of their conduct, they go yet one step further and declare that all who dare adopt any other basis, do so because at their heart they are thieves and robbers.

As the patent law is their mainstay, so professional honor is their great bugbear, which, since they cannot comprehend, they invest with qualities too foul to contemplate, bitterly bemoaning the sacrifices which it exacts, and the depredations which it commits. We must say to our friends, however much we may deprecate the injustice of such dishonorable imputations, we must and will still recognize that there does yet exist such a thing as professional honor. The crown and ornament of our vocation. Does the sun cease to shine because an owl more or less cannot see it? We desire to conclude what we have to say on this subject with an appeal against a too great yielding to the encroachments of the matereal-istic tendencies of our times. Here everywhere its power is felt, but we cannot and will not believe that there are influences and impulses which cannot be treated as things to be coldly analyzed and scientifically dealt with as materials subject to material laws, and material manipulations. And these make life worth living and make men more than splendid and complex machinery. And to these we look with confident expectation and proud hope for the preservation and elevation of professional pride, professional courtesy and professional honor.

